# Why it is Important to Publish

**DOI:** 10.1002/mdc3.14090

**Published:** 2024-05-29

**Authors:** David J. Burn

**Affiliations:** ^1^ Newcastle University Newcastle upon Tyne UK

**Keywords:** publishing, best practice, research misconduct

This article sets the scene for the other contributions that were presented at the Third Movement Disorders Clinical Practice Conference in Cartagena in February 2024. It describes the potential benefits from publishing good quality work and briefly covers less savory publication practices, reinforcing the message that the importance of publishing does not equate to publishing at any cost.

The roots of scholarly scientific publishing may be traced back to 1665 when Henry Oldenburg of the British Royal Society established the *Philosophical Transactions of the Royal Society*. It is said that Oldenburg was motivated by a desire to remove himself as a diplomatic interlocutor between dispersed independent scientists, with whom he communicated individually. In creating the *Transactions*, he effectively generated a public record of knowledge and provided a medium for improved communication. We now take these purposes almost for granted in the numerous journals that exist today.

There are many reasons to publish, and individual motivation and priorities will vary from individual to individual, and in each individual over time. Dissemination and impact, however, must be regarded as paramount, with the aim of producing societal benefit. Discoverability, contributing to the records of research in the relevant field, and preventing duplication of effort are also important; the latter is not the same as replicating scientific findings, which remains a worryingly difficult objective to achieve for many studies. Whilst not always regarded as a benefit by authors, thoughtful and constructive peer review brings an important element of quality control and feedback in the publication process. Many colleagues will publish because a strong curriculum vitae can lead to promotion, or other career advancement. Refreshingly, many higher education institutes have signed up to Declaration on Research Assessment (DORA), and the responsible use of research metrics. This de‐emphasizes the need to chase so called “high impact factor” journals and places more focus on the impacts of the work.

The range of possible publications is considerable and offers the opportunity to publish to nearly all movement disorder specialists, at all points on the clinical‐academic spectrum, and from any discipline. Options include novel clinical observations, a viewpoint, a hypothesis, review, research study and comment on other publications (usually in the form of a letter). Irrespective of the format chosen, it is vital to try to write well. There are many articles available that provide authors with key principles and guidance to clear writing. In this regard, the “ABC of authorship” is worth remembering: Be Accurate, Brief and Clear. There are many resources available online and in the literature that provide advice when writing a scientific paper; a good starting point is: https://www.nature.com/nature-portfolio/for-authors/write.

Tips that I have acquired over the years include the following. Know what you are trying to say before you start writing. This seems self‐evident, but always keeping the primary aim of the publication in mind will make for a more focussed manuscript. Aim for a concise and catchy article title; reviewers and editors like this, although the title shouldn't end up being cryptic. Always aim to generate short, clear sentences. When you are writing imagine your article is to be understood by non‐experts in the field. Don't make the introduction and discussion longer than necessary. Both should draw upon selected and relevant literature to provide context. In effect, the introduction is used to say why you did what you did, whilst the discussion summarizes and interprets the findings and puts them in a broader context. Utilize internal peer review and “critical friends” before submission, particularly if you are relatively new to the publication process. If you are uncertain as to the importance or significance of your proposed report, don't be afraid to ask more experienced colleagues, in other counties if necessary. These same colleagues may even be prepared to look over the grammar and spelling in your draft paper, if English is not your first language. Be ambitious (but also realistic) in your target journal choice. Don't be dispirited by rejection; instead try to learn from and adapt your publication in the light of the peer review comments received and carefully reconsider your target journal options.

Along with the importance, and what is widely perceived as an increasing pressure to publish (which may be self‐generated, from an institution, or other source), come potential negative aspects. These include low quality outputs, research misconduct and predatory publishing practices.

It is worth bearing in mind that only 45% of articles published in 4500 top scientific journals are cited within 5 years of publication.^1^ “Unethical practices” include the salami slicing of data, duplicate publications and dubious authorship justification. Salami slicing breaks the outputs of a study up into smaller elements to produce more publications but loses giving an overall picture of the study data. Duplicate publications target different journals with what is essentially the same data, presented in minimally different ways. Dubious authorship justification may occur, for example, where there is a very hierarchical department. In such cases the head of the unit may expect to be included as an author, even if they have contributed nothing to the publication.

Research misconduct includes the falsification of data and plagiarism.^2^ Most reputable journals will often run software programs to check for both. This may include looking at blots and scans for evidence of “doctoring” of the images, as well as “reading” text to look for excessive similarity with publications elsewhere in the literature. The use of artificial intelligence (AI) in generating publications may be regarded as a two‐edged sword. And like it or not, AI is here to stay and will only become more sophisticated in future. For an author whose primary language is not English, AI can be helpful to improve the quality of the text. The “acid test” of course, is that the data or observations being presented are the work of that author. Sadly, predatory journal practices, which subvert the traditional peer‐reviewed system for financial gain are reported to be increasing.^3^, ^4^

To conclude, publishing high quality work is ultimately for societal benefit, although there are many other associated advantages. Writing a well‐constructed and clear article is a validated and accepted way of communicating with one's colleagues in an engaging way Unfortunately, academic pressure (usually for individual and/or institutional advancement/gain) has led to a rise in unethical practices and research misconduct which we must all be aware of and collectively seek to stamp out. Finally, irrespective of background and experience, do not be afraid to ask for help at any stage of the publication process: the earlier the better (Video 1).

**Video 1:** 2024 MDCP conference recording.

## Disclosures

I declare that there are no conflicts of interest relevant to this work and that there are no additional disclosures to report.
